# The Effects of Reading Engagement on Older Adults’ Resource Allocation in Reading and Cognition

**DOI:** 10.1093/geroni/igaa057.1181

**Published:** 2020-12-16

**Authors:** Giavanna McCall, Sueyoun Hwang, Takudzwa George, Xiaomei Liu, Elizabeth A L Stine-Morrow

**Affiliations:** 1 University of Illinois at Urbana-Champaign, Urbana, Illinois, United States; 2 Beckman Institute, Urbana, Illinois, United States; 3 University of Illinois, Urbana, Illinois, United States

## Abstract

With aging, print exposure has been shown to predict effective resource allocation during reading, as well as to account for growth in certain abilities. To test the hypothesis that literacy engagement has causal effects on reading processes and the cognitive abilities on which they depend, we developed a home-based literacy intervention administered via iPads. Participants (64% female; 60-79 years of age; < 10hrs/week of reading at baseline) were randomly assigned to an 8-week reading intervention group (developed in collaboration with a Champaign Library adult literacy specialist) or to a puzzle control, both of which involved ~8hrs/week of activity engagement. At pretest and posttest, participants performed a self-paced reading task to assess resource allocation to word and conceptual processing, and completed a battery of cognitive measures assessing episodic memory, working memory, and verbal fluency (alphas ≥ .79). Based on one-tailed tests, results showed greater positive change in working memory, t(57)=1.69, p<.05, for the literacy group relative to the control, and a marginally significant difference in change in episodic memory, t(57)=1.62, p<.06, but not in verbal fluency, t< 1. In addition, there was a significant difference between conditions in change in resource allocation to conceptual processing, t(53)=1.75, p<.05. Changes in working memory and conceptual processing were positively correlated (r=.27, p=.02). These findings suggest that reading engagement may be beneficial for older adults’ growth in fluid ability, which impacts reading processes, so as to create a “virtuous spiral” of resilience between literacy engagement and ability.

